# The Role of the Cerebellum in Swallowing

**DOI:** 10.1007/s00455-021-10271-x

**Published:** 2021-03-06

**Authors:** Ayodele Sasegbon, Shaheen Hamdy

**Affiliations:** Gastrointestinal (GI) Sciences, Faculty of Biology, Medicine and Health, Division of Diabetes, Endocrinology and Gastroenterology, School of Medical Sciences, University of Manchester, Salford Royal Hospital (part of the Manchester Academic Health Sciences Center (MAHSC)), Salford, UK

**Keywords:** Cerebellum, Swallowing, Dysphagia, Neurostimulation

## Abstract

Swallowing is a complex activity requiring a sophisticated system of neurological control from neurones within the brainstem, cerebral cortices and cerebellum. The cerebellum is a critical part of the brain responsible for the modulation of movements. It receives input from motor cortical and sensory areas and fine tunes these inputs to produce coordinated motor outputs. With respect to swallowing, numerous functional imaging studies have demonstrated increased activity in the cerebellum during the task of swallowing and damage to the cerebellum following differing pathological processes is associated with dysphagia. Single pulses of transcranial magnetic stimulation (TMS) have been applied to the cerebellum and have been shown to evoke motor responses in the pharynx. Moreover, repetitive TMS (rTMS) over the cerebellum can modulate cerebral motor (pharyngeal) cortical activity. Neurostimulation has allowed a better understanding of the connections that exist between the cerebellum and cerebral swallowing motor areas in health and provides a potential treatment for neurogenic dysphagia in illness. In this review we will examine what is currently known about the role of the cerebellum in the control of swallowing, explore new findings from neurostimulatory and imaging studies and provide an overview of the future clinical applications of cerebellar stimulation for treating dysphagia.

## Swallowing

The act of swallowing occurs so often and without complication as to be almost mundane in the lives of most people. It is a means by which organisms, including humans, can ingest fluids and food in order to maintain their natural physiological and biochemical functions. In addition to the purely functional, the acts of eating and drinking play a social role, acting to bond individuals together. It is for this reason that any impairment of the ability to swallow leads to a large reduction in a person's perceived quality of life [1, 2]. Traditionally, a normal swallow was viewed as being composed of three clearly demarcated phases; an oral phase, a pharyngeal phase and an oesophageal phase [3]. The oral phase of swallowing involves the processing and transportation of ingested food or fluids. Following processing using the teeth (in the case of foods) and tongue, boluses are transported into the pharynx where the pharyngeal phase of swallowing begins [3]. This phase of swallowing involves transformation in addition to transportation. The pharynx must be made safe for swallowing by the closure of the epiglottis so as to prevent aspiration before boluses are propelled towards the upper oesophageal sphincter and into the oesophagus [3]. The oesophagus constitutes the last phase of the journey of a bolus before it reaches the stomach [3]. However, the traditional stark separation between the component phases of a normal swallow have been eroded in modern approaches towards swallowing [3]. Instead of three phases swallowing is thought to be composed of two phases, an oropharyngeal and oesophageal phase, or a single blended oropharyngeal oesophageal phase [3]. This more modern conception of swallowing is informed by the fact that each phase of the swallowing process relies upon the preceding phase to function normally and prevent dysfunction.

The process of swallowing is complex, involving numerous muscles of the face, pharynx and oesophagus. These muscles are controlled by swallowing centres distributed through the brainstem, the cerebral cortex and in the cerebellum. The term swallowing centres is used to refer to any discrete group of neurones which act to initiate and modulate the process of swallowing [4]. As with any complex process, there are multiple points during the execution of a swallow where dysfunction can occur. The breakdown of the normal process of swallowing is called dysphagia [5–7]. Various diseases can cause disruption to normal swallowing, resulting in dysphagia which has the potential to cause malnutrition, dehydration and aspiration pneumonia, all of which lead to significant mortality and morbidity [8].

The afferent nerves of the head and neck which carry sensory information from the oral cavity, pharynx and oesophagus to the central nervous system (CNS), and the efferent nerves which carry information from the CNS to the muscles involved in swallowing, have been well studied and their roles increasingly understood for over a century [9]. However, understanding the way in which the CNS coordinates afferent inputs and efferent outputs during a successful swallow has accelerated in the last two decades due to the advent of new imaging and brain stimulation technologies. These have transformed our understanding of the CNS control of swallowing, particularly in humans.

## Central Nervous Processing of Swallowing

### Cortical and Brainstem Swallowing Centres

Afferent neurons from the upper gastrointestinal tract provide information to the nucleus tractus solitarius (NTS), a sequence of closely interlinked sensory nuclei in the medulla [10]. Together these cell bodies form a sensory relay. Invasive studies using electrodes to stimulate specific regions within the brain in an attempt to initiate the process of swallowing have identified the NTS as the region where swallowing begins [11, 12]. Animal studies have shown targeted lesions to the NTS prevent electrical signals from the superior laryngeal nerve (SLN) from initiating the involuntary phase of swallowing [10]. Furthermore, in animal studies involving lesions to the SLN, similar deleterious effects to those that occurred following NTS damage were seen [13]. The sensory NTS then communicates with motor nuclei within the medulla around the nucleus ambiguus (NA) [14]. This network of interneurons and nuclei orchestrate the highly complex sequence of muscular contractions required for swallowing. The entire interconnected sensory and motor relay in the medulla oblongata is called the central pattern generator (CPG) [7, 10]. Although the CPG functions as a single cohesive unit there are in fact two mirrored CPGs on the left and right sides of the medulla connected via communicating inter-neurones. Each half of the CPG receives neuronal impulses from the ipsilateral side of the oral cavity and pharynx [10]. Medullary swallowing centres are modulated by input from higher cortical centres. These higher ‘centres’ are primarily composed of the representations of the muscles and sensory organs of the oropharynx on the motor and sensory cortices bilaterally [3]. Studies have shown there is a degree of asymmetry over cortical swallowing centres [15]. In essence, one hemispheric motor swallowing ‘centre’ is more active than the other [15]. Over the cerebral motor cortex, the hemispheric oropharyngeal motor representation with the higher basal neuroelectrical level of activity is termed the ‘dominant’ motor cortical centre, while its contralateral companion is termed the ‘non-dominant’ centre [16]. In studies utilising transcranial magnetic stimulation (TMS) the dominant hemispheric motor centre is defined as the area with the lower resting motor threshold (RMT) [16]. In addition to swallowing centres in the medulla and cerebral cortex, invasive animal [17] and human functional imaging [18, 19] studies have shown the involvement of other subcortical regions in the control of swallowing. These include the thalamus, pons, insular cortices, cingulate gyrus and cerebellum [18–20].

### The Cerebellum

The cerebellum is a part of the brain located within the posterior cranial fossa inferior to the occipital lobes of the cerebral cortex [21, 22]. It is a bi-hemispheric structure broadly akin to the cerebral cortex [21, 22] with its hemispheres being connected by a central region called the vermis. The cerebellum is also divided horizontally by two fissures, the primary and horizontal fissures [21]. It occupies less space than the cerebral cortex but contains a large amount of grey matter surrounding a central white matter core. In actuality, despite its position and unassuming organisation, the cerebellum contains the majority of neurones within the brain [21–23]. It attaches directly to the brainstem through the superior, middle and inferior peduncles [22]. Neuroanatomically the cerebellar cortex comprises three neuronal layers, the molecular, purkinje and granular layers [21, 23]. The neuronal components of these layers are highly conserved and repeated across both cortices of the cerebellum [23–25]. Purkinje cells are connected to deep cerebellar nuclei such as the dentate and fastigial nuclei which lie beneath the cerebellar hemispheres [23]. These are themselves connected to the motor cortices [24, 26, 27]. Impulses arrive in the cerebellum either through ‘climbing fibres’ from the contralateral inferior olivary complex (IOC) in the medulla oblongata, or via mossy fibres which receive information from various locations including the pontine reticular formation and pontine nuclei. The IOC is closely involved in the regulation and control of movement [21–23].

The cerebellum has long been known to be involved with the process of movement. Although movements are initiated in the cerebral cortex, the cerebellum plays a key role in ensuring any muscular activity is accurate, smooth and co-ordinated [23–25]. Neurones from the motor and sensory cortices combine to form the cortico-olivocerebellar tract. These then synapse with the IOC bilaterally. The cortico-olivocerebellar tract along with the reticulocerebellar and pontocerebellar tracts carry information pertaining to the initiation of movements and ongoing moments [23, 24]. While lesions of motor areas of the cortex can result in paralysis, lesions of the cerebellum result in tremulous, uncoordinated and inaccurate movements [23, 24].

### The Cerebellum and Neurostimulation

**Fig. 1:**
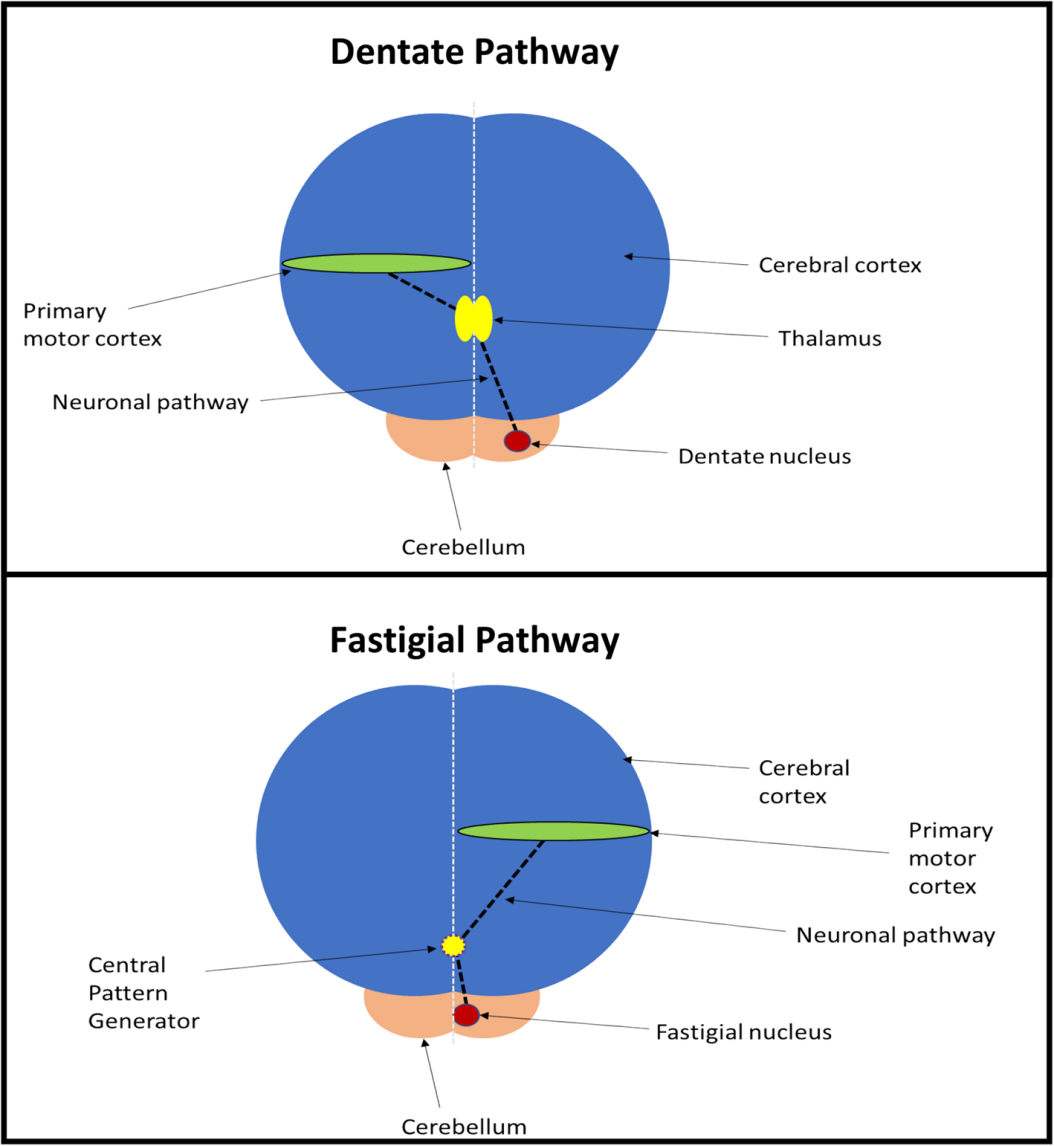
Dentate and potential fastigial cerebello-cortical motor pathways

As stated previously, pyramidal neurones in the cerebellar cortex connect to and communicate with deep nuclei within the cerebellum [23, 28]. These in turn communicate with cerebral motor cortical areas [23]. The most well studied cerebello-cortical pathway involves the dentate motor nuclei located in both cerebellar hemispheres [29–31]. When stimulated, these communicate with the thalamus before crossing over to terminate in the contralateral motor cortical hemisphere [23] (Fig. 1).

Early invasive animal studies showed electrical stimulation of the cerebellar cortex caused inhibition of subsequent motor cortical activity. One such study was performed by Yamamoto et al. in cats. Forty-eight cats had their cerebellums exposed by craniotomies and had electrodes implanted into their cerebellar nuclei. Interestingly, both excitatory and inhibitory responses were observed in neurones in layers 2 and 3 of the brain [32]. This observation of mixed excitatory and inhibitory cerebello cerebrocortical communication was later observed in primates [26]. Studies such as these, set the foundation for non-invasive human studies.

In 1991, Ugawa et al. delivered electrical impulses to the cerebellums of 10 healthy participants using scalp electrodes attached over the posterior fossa [31]. They found that conditioning cerebellar electrical impulses delivered between 5 and 8 ms before a secondary TMS impulse was administered over the dorsal interosseous region of the cerebral motor cortical hemisphere, caused a reduction in cortical MEP amplitudes [31]. Furthermore, this suppressive effect was noted to occur only at cerebellar stimulation intensities close to the cerebellar resting motor threshold (RMT). In 1995, a similar study by Ugawa et al. using TMS to administer both conditioning and secondary stimulation, confirmed these findings of stimulation intensity dependant cerebellar motor cortical inhibition [30]. Subsequently a 2001 study by Pinto et al. also utilising conditioning and secondary TMS pulses confirmed the findings of the earlier two studies [29]. When viewed together, these studies show that stimulation of the cerebellum whether by electrical or magnetic means causes cerebral motor cortical inhibition.

**Fig. 2:**
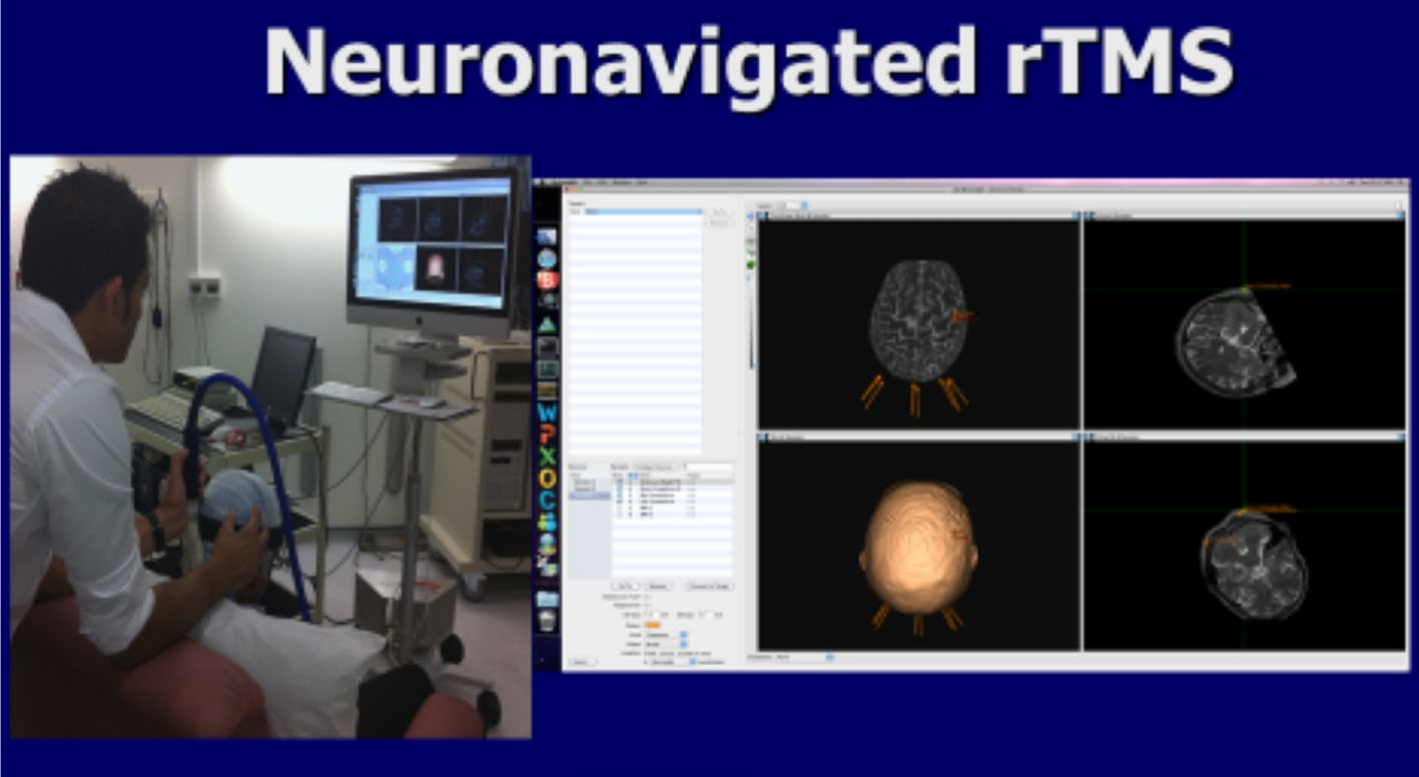
Cerebellar rTMS being delivered guided by MRI frameless stereotaxy (Image from GI Sciences University of Manchester dysphagia research group)

Within the swallowing motor system, the first neurostimulation study in humans to show stimulation of the cerebellum leads to cerebral swallowing motor area changes was performed by Jayasekeran et al. in 2011 (Table 1). In this healthy participant study, single pulse TMS applied to the cerebellar hemispheres or vermis (Fig. 2) resulted in detectable pharyngeal MEPs (the first time this had been observed). Furthermore, and most importantly, TMS applied in a paired pulse manner resulted in increases to cerebral cortical pharyngeal area MEP amplitudes [33]. The greatest faciliatory effect was observed when (hemispheric) cerebellar conditioning pulses preceded subsequent cortical TMS pulses by 50 to 200 ms. This was a surprising finding as previous studies investigating the cortical effects of cerebellar stimulation had always shown inhibition. However, these studies had all utilised cerebellar stimulation targeted at hand motor areas [30, 31]. This discrepancy may be indicative of underlying differences between cerebellar modulation of the limb and swallowing motor systems.

**Table 1:** Table of cerebellar swallowing neurostimulation studies

Authors	Type of study	Participants	Type of neurostimulation	Frequency and duration of neurostimulation	Intensity of stimulation	Outcome measures	Key findings
Jayasekeran 2011 [ 33 ]	Cross over	Healthy 16	rTMS	Arm 1: Paired pulse cerebellar TMS Arm 2: Paired pulse trigeminal nerve stimulation Arm 3: Sham	110% RMT	PMEP amplitude	Cerebellar paired pulse TMS significantly increased cortical pharyngeal area excitability
Vasant 2015 [ 34 ]	Cross over	Healthy 17	rTMS	Arm 1: 600 pulses of rTMS at 1 Hz Arm 2: 250 pulses of rTMS at 5 Hz Arm 3: 250 pulses of rTMS at 10 Hz Arm 4: 250 pulses of rTMS at 20 Hz Arm 5: Sham	Arm 1: 90% cerebellar RMT Arms 2–5: 90% thenar RMT	PMEP amplitude	10 Hz cerebellar hemispheric rTMS significantly increased cortical pharyngeal area excitability
Sasegbon 2019 [ 4 ]	Cross over	Healthy 15	rTMS	Arm 1: 250 pulses of contra-lesional rTMS at 10 Hz Arm 2: 250 pulses of ipsi-lesional rTMS at 10 Hz Arm 3: Sham	90% thenar RMT	PMEP amplitude Swallowing behaviour	Ipsi and contra-lesional cerebellar hemispheric rTMS were significantly better than sham at reversing the suppressive PMEP and disruptive behavioural effects of a cortical virtual lesion There were no differences between the reversal effects of ipsi or contra-lesional cerebellar rTMS
Vasant 2019 [ 42 ]	Single patient cross over study	1 patient with stroke affecting cerebellum and medulla *(patient received sham and real rTMS on different days)*	rTMS	Active stimulation: 250 pulses of contra-lesional rTMS at 10 Hz Sham	90% thenar RMT	PMEP amplitude VFS PAS score	Increase in cerebral pharyngeal motor cortical area excitability following active cerebellar rTMS Improvement in VFS PAS following active rTMS compared to sham
Sasegbon 2020 [ 16 ]	Cross over	Healthy 13	rTMS	Arm 1: 250 pulses of at 10 Hz over the right hemisphere Arm 2: 250 pulses of at 10 Hz over right and left hemispheres	90% thenar RMT	PMEP amplitude Swallowing behaviour	Bi-hemispheric cerebellar rTMS was significantly better than uni-hemispheric cerebellar rTMS at: Causing pharyngeal motor cortical excitation Reversing the suppressive PMEP and disruptive behavioural effects of a cortical virtual lesion
Sasegbon 2020 [ 41 ]	Cross over	Healthy 12	rTMS	Arm 1: 250 pulses at 10 Hz Arm 2: Sham	90% thenar RMT	PMEP amplitude Swallowing behaviour	Cerebellar vermis rTMS significantly suppressed cerebral pharyngeal motor cortical excitability and disrupted swallowing behaviour

*PAS* Penetration aspiration score, *PMEP* Pharyngeal motor evoked potential, *RMT* Resting motor threshold, *rTMS* Repetitive transcranial magnetic stimulation, *VFS* Videofluoroscopy

The first repetitive TMS (rTMS) study in the cerebello-cortical swallowing system was performed by Vasant et al. in 2015 [34]. Although several studies had been done using high-frequency rTMS to stimulate various areas of the cerebral cortex, the cerebellum had not been studied. Vasant et al. sought to investigate the cerebral pharyngeal motor cortical effects of a range of frequencies of cerebellar rTMS [34]. The interventional arms were: 1 Hz rTMS (known to be inhibitory when applied to cerebral motor cortical areas), 5 Hz rTMS (known to be excitatory when applied to cerebral motor cortical areas), 10 Hz rTMS, 20 Hz rTMS and sham rTMS. Ten Hz cerebellar rTMS was found to significantly increase the excitability – measured via MEP amplitudes – of the pharyngeal area of the motor cortex [34] with the excitatory effect observed to last for over 60 min [34]. Once more, just as in the preceding study [33], cerebellar stimulation led to cortical excitation rather than suppression.

Following the study findings above, in 2019 Sasegbon et al. performed an experimental cortical ‘virtual lesion’ reversal study investigating the neurophysiological and swallowing behavioural effects of cerebellar rTMS applied over the cerebellar hemispheres [4]. The cortical ‘virtual lesion’ protocol was developed by Mistry et al. in 2007 [35] and involves the application of 10 min of 1 Hz (suppressive) rTMS to the cerebral pharyngeal motor hemisphere with the lowest RMT (the ‘dominant’ hemisphere). It is used as a hemispheric “stroke” model in healthy participants to test the effects of novel neuro-stimulatory interventions [36]. In the Sasegbon study, 10 Hz cerebellar rTMS applied to the right and left hemispheric pharyngeal areas was able to fully reverse the suppressive pharyngeal MEP and disruptive swallowing behavioural effects of a ‘virtual lesion’. Interestingly, reversal was seen to occur regardless of whether cerebellar rTMS was applied ipsi or contralaterally to the cerebral hemispheric location of the ‘virtual lesion’. This ipsilateral effect was unexpected as the well-known dentate cerebello-cortical neuronal pathway described above indicates that cerebello-cortical connectivity occurs contralaterally. Unfortunately, no post cerebellar neurostimulation functional imaging studies have been performed which means the precise nature of this ipsilateral effect is unknown. However, potential explanations for this include an ipsilateral cerebello-cortical neuronal pathway via fastigial nuclei within the cerebellum. These send out efferent projections to the brainstem which terminate in the area around the NA [27] (Fig. 1). This constitutes much of the CPG along with the NTS. The CPG in turn communicates with cortical motor swallowing areas bilaterally [23]. Alternately, the observed ipsilateral excitatory effect may be due to cerebral inter-hemispheric motor communication from the contralateral pharyngeal motor area [37–40]. The excitatory findings of the Sasegbon study were confirmed in 2020 by another study from the same group [16]. In this study the motor cortical and swallowing behavioural effects of uni-hemispheric and bi-hemispheric cerebellar rTMS were explored. In the study, bi-hemispheric rTMS was significantly more excitatory and had greater reversal effects than uni-hemispheric cerebellar rTMS [16].

Recently a cerebellar neurophysiological study by the Hamdy group has shed more light on the effects of cerebellar rTMS on cerebral motor cortical activity and swallowing function. In this experiment, Sasegbon et al. studied the motor cortical and behavioural effects of cerebellar rTMS delivered over the vermis instead of the hemispheres [41]. They found that vermis cerebellar rTMS resulted in suppression of pharyngeal cerebral motor cortical areas and disruption to swallowing behaviour. This suppressive effect can be seen as being similar to the suppression previously reported when cerebellar TMS is applied over limb motor areas. Furthermore, this indicates the presence of site specificity of neuro-stimulatory effect and the possible existence of a different population of neurones within the cerebellar vermis with different cerebello-cortical projections. New functional imaging studies will need to be performed to cast light on this new and rapidly changing area.

**Fig. 3:**
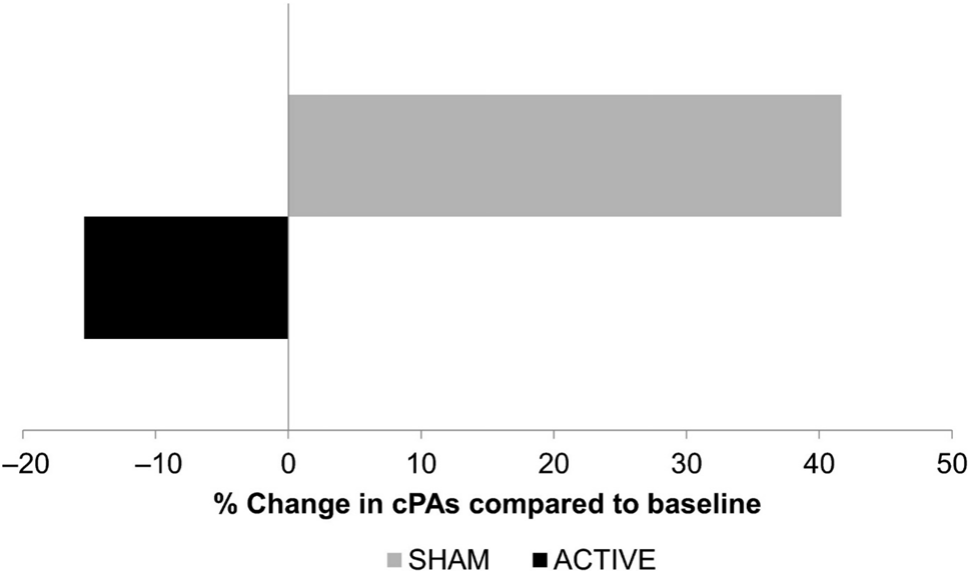
The effects of active and sham contra-lesional cerebellar rTMS on cumulative aspiration scores (lower scores indicate more normal swallowing function). [34]

Clinically, a single study has been published which used cerebellar targeted rTMS to ameliorate post-stroke dysphagia (PSD). Published in 2019, Vasant et al. extensively studied a single patient with a brain stem stroke in a cross-over designed experiment [42]. They found that cerebellar rTMS was able to increase cerebral motor cortical PMEP amplitudes and lead to improvements in videofluoroscopy (VFS) Penetration Aspiration Scores (PAS) (Fig. 3). However, while promising, little can be extrapolated from this study because the use of a single patient means its results cannot be subjected to statistical analysis and causality cannot be determined.

### Swallowing and the Cerebellum

Invasive animal studies using microelectrodes have shown that the application of electrical impulses to the cerebellar vermis of cats is able to induce swallowing behaviour [25]. Subsequent, feline studies by Reis et al. Martner et al. and Berntson et al. showed electrical stimulation of cerebellar fastigial nuclei results in observable changes to feeding behaviour [43–45]. In 1974, a study by Ball et al. in rats also demonstrated that gnawing and swallowing behaviour could be elicited by delivering electrical stimulation to the cerebellar vermis [46].

In humans, functional imaging techniques including positron emission tomography (PET) and functional magnetic resonance imaging (fMRI) have shown that the cerebellum is bilaterally activated during swallowing [18, 47–49].

**Fig. 4:**
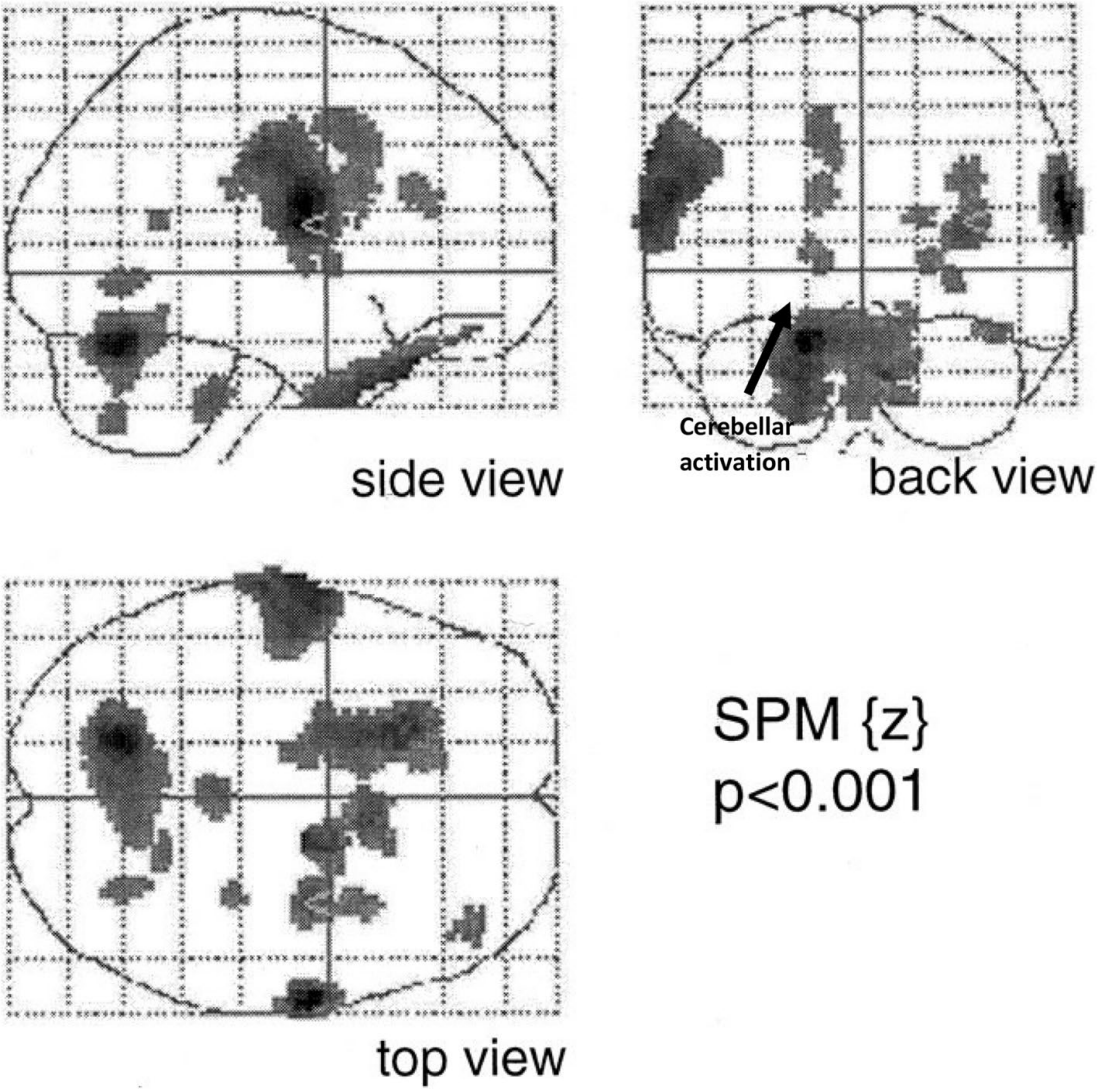
Areas of increased blood flow following swallowing task. [51]

One such fMRI study was performed in 1999 by Mosier et al. [50]. In the study, eight participants were asked to perform a sequence of dry swallows; swallows of small 3 ml water blouses and finger taps [50]. During swallowing there was bilateral activation of the primary motor cortex, sensory cortex and supplemental motor areas. Increased activity was also observed to occur over the cerebellar hemispheres. That same year, a PET study by Hamdy et al. [51] of eight healthy participants additionally showed activation of the cerebellum during swallowing (Fig. 4). In that study, cerebellar activation was seen to occur in all eight participants over the cerebellar hemispheres and the cerebellar vermis, albeit with noticeably stronger activation in the left cerebellar hemisphere [51]. Another fMRI study was performed by Suzuki et al. in 2003 [18]. The study had a slightly larger sample size than the previous studies with eleven healthy participants. In addition to the activation of the aforementioned motor cortical areas, the cerebellum was observed to display a bi-hemispheric increase in activity during swallowing [18]. It is interesting to note that in this study, in contrast to the earlier Hamdy et al. study, only the lateral aspects of the cerebellar hemispheres showed increased activity. The cerebellar vermis was unaffected. Moreover, in 2001 and 2020, fMRI cerebellar mapping studies by Grodd et al. and Bolliat et al. have showed that the lips and tongue (both involved in swallowing) are represented over the cerebellar hemispheres and vermis [52, 53].

Some functional imaging studies have highlighted greater swallowing related activation of the left cerebellar hemisphere compared to the right [48, 49]. The significance of this finding is currently unknown but if investigated and confirmed, future studies may indicate the existence of cerebellar swallowing area hemispheric asymmetry in a manner reminiscent of the known phenomenon of cortical hemispheric pharyngeal area asymmetry [15].

Understanding the topographic organisation of the cortical and cerebellar motor homunculi may go some way towards explaining the findings from the swallowing functional imaging studies mentioned above. The cerebral motor cortices are arranged such that there are discrete areas controlling the various muscles and muscle groups [54]. However, the cortical area responsible for the control of a specific muscle does not correspond to the volume of that muscle [54]. Rather, muscles requiring a greater degree of fine control occupy more cortical space [54]. Were the cortical areas used to construct a humanoid figure (or homunculus), the figure would appear to be distorted with large lips, hands, feet and tongue but with spindly limbs. This hypothetical distorted figure, known about for eight decades [55], is the cerebral motor cortical homunculus [56]. Although it was not always thought to be the case [57], muscle groups also appear in discreet locations within the matter of the cerebellum. The cerebellum possesses its own lesser known more recently discovered motor homunculus. As is often the case, animal studies provided the foundation of knowledge upon which later human work was built. These studies were invasive, utilised decerebrate animals, and involved implanted micro electrodes or the use of neuro-tropic viruses [58–60]. Most of this early work was focused on identifying the locations of limb muscles. What they found was the cerebellar homunculus is completely unlike the better known cerebral cortical homunculus. While each cerebral cortical hemisphere possesses a single motor homunculus, in the cerebellum, each cerebellar hemisphere possesses three motor homunculi [61]. Two arise on the superior aspect of the cerebellum and one on its inferior aspect [61, 62]. Furthermore, there is evidence to suggest another homunculus exists along the cerebellar vermis or midline [61, 62]. Were it to be modelled, the cerebellar homunculus would look akin to a spider. It is perhaps, therefore, less surprising that swallowing functional imaging studies have identified three areas of increased activity within the cerebellum. Namely, over both hemispheres and the cerebellar vermis.

With regards to whether the cerebellum influences the oral, pharyngeal or oesophageal stages of swallowing, fMRI mapping studies over the human cerebellum have shown that the lips and tongue are represented over the cerebellum [53]. Furthermore, as stated earlier, single pulse TMS has shown that pharyngeal motor evoked potentials (PMEPs) can be evoked following cerebellar stimulation [33]. When assessed in combination, the results of these studies show the cerebellum acts, in some way, to modulate the activity of muscles within the oral and pharyngeal phases of swallowing. There have been no studies showing the oesophagus is represented topographically within the cerebellum. Furthermore, as stated in the article, electrical stimulation of the brains of animals including cats and rats [43, 46] have shown that it is possible to induce visible swallowing and chewing behaviour. This observation further strengthens the case that the cerebellum is a part of the swallowing motor system. In humans, rTMS stimulation of the cerebellum has been shown to cause measurable changes in cerebral motor cortical PMEP amplitudes [16]. However, TMS or rTMS has not been shown to be able to provoke swallowing in the same manner as electrical stimulation in animal studies. One potential reason for this is that modern rTMS protocols are limited with respect to the energies they impart to targeted brain tissue to reduce any risk of inducing seizure activity [63]. Were these safety limits breached it is conceivable that high intensity rTMS will be able to induce frank swallowing behaviour.

### Cerebellar Pathology and Dysphagia

Diseases which affect the cerebellum have been demonstrated to cause dysphagia [42, 64, 65]. This is likely due to disruption of the motor modulatory influence the cerebellum has on cerebral motor cortical areas [24]. However, the extent to which cerebellar lesions can cause dysphagia is seemingly disease dependant. Case studies in patients with Chiari malformations have shown dysphagia occurs in up to 47% of cases [66, 67]. Chronic neurodegenerative conditions such as cerebellar ataxia [65] multiple system atrophy (MSA) [68, 69] and multiple sclerosis (MS) [64] have also been shown to be strongly associated with dysphagia. In cerebellar ataxia, a 2020 questionnaire-based study in 119 patients by Ronnefarth et al. found 17% of patients to have dysphagia based on the swallowing-related quality of life (Swal-QOL) assessment tool [70]. Dysphagia is a common occurrence in MSA occurring in up to 73% of patients [71]. Studies have shown that the cerebellar variant of MSA causes dysphagia because of incoordination of oropharyngeal muscular contractility [72]. MS is also known to affect the cerebellum and can cause dysphagia [64]. In 1999 and 2010, studies by Wiles et al. and Poorjavad et al. showed that patients with dysphagia were significantly more likely to have neurological impairment affecting their cerebellums than those without dysphagia [73, 74].

Several neurological conditions are known to cause dysphagia as well as being known to cause cerebellar atrophy including: Alzheimer’s disease (AD) [75, 76], progressive supernuclear palsy (PSP) [77, 78], amyotrophic lateral sclerosis (ALS) [79–81] and frontotemporal dementia (FTD) [82, 83]. However, the precise ways in which cerebellar atrophy may cause dysphagia in all these conditions has not been well studied. Furthermore, in all of the conditions described, dysphagia is also caused by non-cerebellar causes. Examples of this include the dysphagia which occurs in AD [83]. Neurological degeneration in AD is known to affect several parts of the swallowing motor pathway including the cerebellum [76]. As a result, the amount to which the cerebellum contributes to any dysphagia that emerges is difficult to quantify. In addition, patients with AD often develop cognitive impairment [3] which is independently associated with dysphagia [3]. Another example is in PSP associated dysphagia. Dysphagia can occur in up to 83% of patients with undifferentiated PSP [71]. In addition, dysphagia is also a feature of the cerebellar ataxic variant of PSP [84]. However, studies have shown that dysphagia occurs largely due to spasticity of the muscles which make up the oropharynx [78]. Although incoordination, a hallmark of cerebellar impairment [84], occurs [78], it is unclear to what degree this plays a part in any observed dysphagia. Furthermore, cognitive impairment occurs in PSP [78].

Despite these findings, evidence from stroke studies are less clear regarding dysphagia occurring secondary to cerebellar pathology. In 2011, a meta-analysis by Flowers et al. which explored the risk of dysphagia occurring following strokes in specific brain regions, did not show any dysphagia occurring in patients with isolated cerebellar strokes [85]. However, damage to the structures of the brainstem including the medulla and pons did cause dysphagia [85]. Following this, in 2016, a study by Dehaghani et al. in 116 stroke patients, did not find cerebellar damage to be significantly associated with dysphagia [86]. More recently, studies in 2018 and 2019 by Mo et al. and Fernandez-Pombo et al. have not shown any significant association between cerebellar lesions and dysphagia [87, 88].

The contrasting findings from the studies above suggests that discrete cerebellar damage in isolation is not a direct cause of dysphagia, rather cerebellar damage in addition to damage to brainstem structures causes dysphagia. The cerebellum’s role is the modulation of motor activity; therefore, it may be the case that isolated damage may cause an element of incoordination of swallowing muscular activity but may not be sufficient to cause dysphagia. It is likely that the cerebellum does not form a part of the primary motor swallowing pathway which exists within the brainstem but acts to influence motor cortical swallowing centres as a force multiplying adjunct, further enhancing oropharyngeal muscle coordination. An additional piece of evidence in support of this supposition, can be found in the 2019 cerebellar rTMS stroke study by Vasant et al. [42]. In this single case study, the patient with dysphagia had a stroke affecting the cerebellum and brainstem [42] suggesting the need for dual lesion disease. Another possible reason as to why damage to the cerebellum alone may not result in overt dysphagia is the fact that the representations of swallowing musculature occur in multiple locations over the cerebellum. This may mean that damage to a localised part of the cerebellum is less likely to be deleterious and cause dysphagia, compared to a similar lesion in the cerebral cortex. More studies are required in this area so more definitive answers can be provided.

## Conclusion

**Fig. 5:**
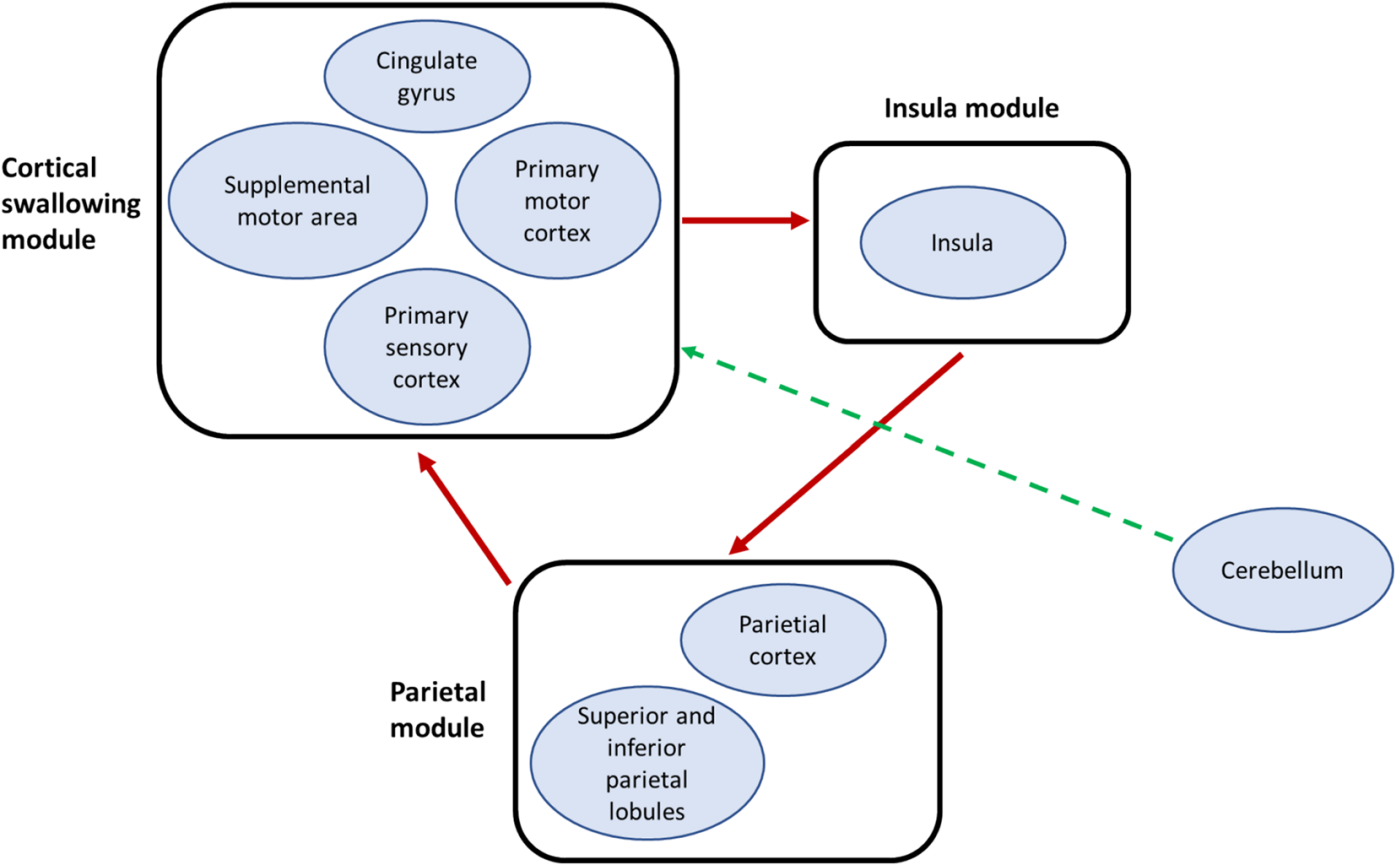
Modular swallowing areas within the brain. Arrows show cortical parietal insula circuit and cerebellar modulation. Image simplified and adapted from [19]

Multiple functional imaging studies show the cerebellum is active during volitional swallowing [18, 50, 51]. Additionally, animal studies have shown that electrical stimulation of the cerebellum can trigger chewing and feeding behaviour in different animal species [43, 46]. Subsequent non-invasive neurostimulation studies in healthy human participants have shown rTMS targeted over the cerebellar hemispheres or vermis can cause cortical pharyngeal area excitation [34] or suppression [41] as well as resulting in changes to swallowing behaviour [16]. When taken together, the narrative these studies give is that the cerebellum is an important part of swallowing motor control. However, studies involving cerebellar pathology have cast some doubt on the importance of the cerebellum in the neurological co-ordination of swallowing. Stroke studies have shown isolated and discrete cerebellar lesions are unlikely to directly lead to dysphagia [85]. These seemingly disparate and contrasting strands of evidence can perhaps be interwoven by reference to studies indicating the neurophysiological control of swallowing is driven not, as may be expected, by a single large circuit encompassing all swallowing related brain areas, but instead by multiple modular swallowing circuits each interacting with and running parallel to each other [19]. The cerebellum influences other swallowing circuits including, for example, a circuit composed of the primary motor, supplementary motor and primary sensory cortical areas and the cingulate gyrus [19] (Fig. 5). The manner of this influence is not just thought to be fine tuning of distantly initiated motor activity [89], but the generation of a hypothetical internal model of motor activity immediately prior to movements being initiated so as to allow movements to be compared and adjusted against this internal ideal [19, 90]. Therefore, it may be that the cerebellar part of the modular circuit is not as essential to the initiation of swallowing as for example circuits containing the primary motor areas or brainstem structures. Instead, isolated damage to the cerebellum may more likely result in relative incoordination as opposed to complete cessation of a swallow and clinically relevant dysphagia. Despite this, the cerebellum, given its modulatory effects on the cortex, offers an exciting new avenue for neuro-stimulatory treatments which need to be studied in further detail. More and larger functional imaging and neurostimulation studies are required in this field so as to provide answers to bridge these gaps in our knowledge.

## References

[1] Eslick G, Talley J (2008). Dysphagia: epidemiology, risk factors and impact on quality of life - a population-based study. Aliment Pharmacol Ther.

[2] Ney D, Weiss J, Kind A, Robbins J (2009). Senescent Swallowing: Impact, Strategies and Interventions. Nutrition in Clinical Practice.

[3] Sasegbon A, Hamdy S (2017). The anatomy and physiology of normal and abnormal swallowing in oropharyngeal dysphagia. Neurogastroenterol Motil..

[4] Sasegbon A, Watanabe M, Simons A, Michou E, Vasant DH, Magara J (2019). Cerebellar repetitive transcranial magnetic stimulation restores pharyngeal brain activity and swallowing behaviour after disruption by a cortical virtual lesion. J Physiol.

[5] Kuhlemeier K (1994). Epidemiology and Dysphagia Dysphagia.

[6] Malagelada J, Bazzoli F, Elewaut A, Fried M, Krabshuis J, Lindberg G (2007). Dysphagia.

[7] Rofes L, Arreola V, Almirall J, Cabre M, Campins L, Garcia-Peris P (2011). Diagnosis and Management of Oropharyngeal Dysphagia and Its Nutritional and Respiratory Complications in the Elderly. Gastroenterology Research and Practice.

[8] Jaradeh S (1994). Neurophysiology of swallowing in the aged. Dysphagia.

[9] Swanson L (2014). Neuroanatomical Terminology: A Lexicon of Classical Origins and Historical Foundations.

[10] Jean A (2001). Brain Stem Control of Swallowing: Neuronal Network and Cellular Mechanisms. Physiol Rev.

[11] Miller AJ (1972). Characteristics of the swallowing reflex induced by peripheral nerve and brain stem stimulation. Exp Neurol.

[12] Kessler JP, Jean A (1985). Identification of the medullary swallowing regions in the rat. Exp Brain Res.

[13] Wasserman JM, Sundaram K, Alfonso AE, Rosenfeld RM, Har-El G (2008). Determination of the function of the internal branch of the superior laryngeal nerve after thyroidectomy. Head Neck.

[14] Jean A, Kessler JP, Tell F. 1994 Nucleus of the Solitary Tract. 355–69.

[15] Hamdy S, Aziz Q, Rothwell JC, Singh KD, Barlow J, Hughes DG (1996). The cortical topography of human swallowing musculature in health and disease. Nat Med.

[16] Sasegbon A, Smith C, Bath PM, Rothwell J, Hamdy S (2020). The effects of unilateral and bilateral cerebellar rTMS on human pharyngeal motor cortical activity and swallowing behavior. Exp Brain Res.

[17] Bieger D, Hockman CH (1976). Suprabulbar modulation of reflex swallowing. Exp Neurol.

[18] Suzuki M, Asada Y, Ito J, Hayashi K, Inoue H, Kitano H (2003). Activation of cerebellum and basal ganglia on volitional swallowing detected by functional magnetic resonance imaging. Dysphagia.

[19] Mosier K, Bereznaya I (2001). Parallel cortical networks for volitional control of swallowing in humans. Exp Brain Res.

[20] Hockman CH, Bieger D, Weerasuriya A (1979). Supranuclear pathways of swallowing. Prog Neurobiol.

[21] Moore K, Dalley A (2006). Clinically Oriented Anatomy.

[22] Krebs C. Functional Areas: CEREBELLUM http://www.neuroanatomy.ca/functional_areas/cerebellum.html: University of British Columbia; 2006 [

[23] Roostaei T, Nazeri A, Sahraian MA, Minagar A (2014). The human cerebellum: a review of physiologic neuroanatomy. Neurol Clin.

[24] Daskalakis ZJ, Paradiso GO, Christensen BK, Fitzgerald PB, Gunraj C, Chen R (2004). Exploring the connectivity between the cerebellum and motor cortex in humans. J Physiol.

[25] Mussen AT (1930). The cerebellum: The influence of the cortical reactions on the classification and the homology of the lobes and fissures in the cat, monkey and man. Archives of Neurology & Psychiatry.

[26] Holdefer RN, Miller LE, Chen LL, Houk JC (2000). Functional connectivity between cerebellum and primary motor cortex in the awake monkey. J Neurophysiol.

[27] Zhang XY, Wang JJ, Zhu JN (2016). Cerebellar fastigial nucleus: from anatomic construction to physiological functions. Cerebellum Ataxias.

[28] Bostan AC, Dum RP, Strick PL (2013). Cerebellar networks with the cerebral cortex and basal ganglia. Trends Cogn Sci.

[29] Pinto AD, Chen R (2001). Suppression of the motor cortex by magnetic stimulation of the cerebellum. Exp Brain Res.

[30] Ugawa Y, Uesaka Y, Terao Y, Hanajima R, Kanazawa I (1995). Magnetic stimulation over the cerebellum in humans. Ann Neurol.

[31] Ugawa Y, Day BL, Rothwell JC, Thompson PD, Merton PA, Marsden CD (1991). Modulation of motor cortical excitability by electrical stimulation over the cerebellum in man. J Physiol.

[32] Noda T, Yamamoto T (1984). Response properties and morphological identification of neurons in the cat motor cortex. Brain Res.

[33] Jayasekeran V, Rothwell J, Hamdy S (2011). Non-invasive magnetic stimulation of the human cerebellum facilitates cortico-bulbar projections in the swallowing motor system. Neurogastroenterol Motil.

[34] Vasant DH, Michou E, Mistry S, Rothwell JC, Hamdy S (2015). High-frequency focal repetitive cerebellar stimulation induces prolonged increases in human pharyngeal motor cortex excitability. J Physiol.

[35] Mistry S, Verin E, Singh S, Jefferson S, Rothwell JC, Thompson DG (2007). Unilateral suppression of pharyngeal motor cortex to repetitive transcranial magnetic stimulation reveals functional asymmetry in the hemispheric projections to human swallowing. J Physiol.

[36] Michou E, Mistry S, Jefferson S, Singh S, Rothwell J, Hamdy S (2012). Targeting unlesioned pharyngeal motor cortex improves swallowing in healthy individuals and after dysphagic stroke. Gastroenterology.

[37] Mourão LF, Friel KM, Sheppard JJ, Kuo HC, Luchesi KF, Gordon AM (2017). The Role of the Corpus Callosum in Pediatric Dysphagia: Preliminary Findings from a Diffusion Tensor Imaging Study in Children with Unilateral Spastic Cerebral Palsy. Dysphagia.

[38] Murase N, Duque J, Mazzocchio R, Cohen LG (2004). Influence of interhemispheric interactions on motor function in chronic stroke. Ann Neurol.

[39] Takeuchi N, Oouchida Y, Izumi S (2012). Motor control and neural plasticity through interhemispheric interactions. Neural Plast.

[40] Bloom JS, Hynd GW (2005). The role of the corpus callosum in interhemispheric transfer of information: excitation or inhibition?. Neuropsychol Rev.

[41] Sasegbon A, Niziolek N, Zhang M, Smith CJ, Bath PM, Rothwell J (2020). Cerebellum.

[42] Vasant DH, Sasegbon A, Michou E, Smith C, Hamdy S (2019). Rapid improvement in brain and swallowing behavior induced by cerebellar repetitive transcranial magnetic stimulation in poststroke dysphagia: A single patient case-controlled study. Neurogastroenterol Motil.

[43] Reis DJ, Doba N, Nathan MA (1973). Predatory attack, grooming, and consummatory behaviors evoked by electrical stimulation of cat cerebellar nuclei. Science.

[44] Martner J (1975). Cerebellar influences on autonomic mechanisms. An experimental study in the cat with special reference to the fastigial nucleus. Acta Physiol Scand Suppl..

[45] Berntson GG, Potolicchio SJ, Miller NE (1973). Evidence for higher functions of the cerebellum: eating and grooming elicited by cerebellar stimulation in cats. Proc Natl Acad Sci U S A.

[46] Ball GG, Micco DJ, Berntson GG (1974). Cerebellar stimulation in the rat: complex stimulation-bound oral behaviors and self-stimulation. Physiol Behav.

[47] Malandraki GA, Sutton BP, Perlman AL, Karampinos DC, Conway C (2009). Neural activation of swallowing and swallowing-related tasks in healthy young adults: an attempt to separate the components of deglutition. Hum Brain Mapp.

[48] Zald DH, Pardo JV (1999). The functional neuroanatomy of voluntary swallowing. Ann Neurol.

[49] Hamdy S, Rothwell JC, Brooks DJ, Bailey D, Aziz Q, Thompson DG (1999). Identification of the cerebral loci processing human swallowing with H2(15)O PET activation. J Neurophysiol.

[50] Mosier KM, Liu WC, Maldjian JA, Shah R, Modi B (1999). Lateralization of cortical function in swallowing: a functional MR imaging study. AJNR Am J Neuroradiol.

[51] Hamdy S, Rothwell J, Brooks D, Bailey D, Aziz Q, Thompson D (1999). Identification of the Cerebral Loci Processing Human Swallowing With H_2_^15^O PET Activation. J Neurophysiol.

[52] Grodd W, Hülsmann E, Lotze M, Wildgruber D, Erb M (2001). Sensorimotor mapping of the human cerebellum: fMRI evidence of somatotopic organization. Hum Brain Mapp.

[53] Boillat Y, Bazin PL, van der Zwaag W (2020). Whole-body somatotopic maps in the cerebellum revealed with 7T fMRI. Neuroimage.

[54] Wassermann EM, McShane LM, Hallett M, Cohen LG (1992). Noninvasive mapping of muscle representations in human motor cortex. Electroencephalogr Clin Neurophysiol.

[55] Penfield W, Boldrey E (1937). Somatic motor and sensory representation in the cerebral cortex of man as studied by electrical stimulation. Brain.

[56] Roux FE, Niare M, Charni S, Giussani C, Durand JB (2020). Functional architecture of the motor homunculus detected by electrostimulation. J Physiol.

[57] Luciani L (1891). Il cervelletto; nuovi studi di fisiologia normale e patologica.

[58] Sumi T (1969). Some properties of cortically-evoked swallowing and chewing in rabbits. Brain Res.

[59] Bell FR, Lawn AM (1956). Delineation of motor areas in the cerebral cortex of the goat. J Physiol.

[60] Narita N, Yamamura K, Yao D, Martin RE, Sessle BJ (1999). Effects of functional disruption of lateral pericentral cerebral cortex on primate swallowing. Brain Res.

[61] Rijntjes M, Buechel C, Kiebel S, Weiller C (1999). Multiple somatotopic representations in the human cerebellum. NeuroReport.

[62] Schlerf JE, Verstynen TD, Ivry RB, Spencer RM (2010). Evidence of a novel somatopic map in the human neocerebellum during complex actions. J Neurophysiol.

[63] Rossi S, Hallett M, Rossini PM, Pascual-Leone A, Group SoTC (2009). Safety, ethical considerations, and application guidelines for the use of transcranial magnetic stimulation in clinical practice and research. Clin Neurophysiol..

[64] Prosiegel M, Schelling A, Wagner-Sonntag E (2004). Dysphagia and multiple sclerosis. Int MS J.

[65] Abdulmassih EM, Teive HA, Santos RS (2013). The evaluation of swallowing in patients with spinocerebellar ataxia and oropharyngeal dysphagia: A comparison study of videofluoroscopic and sonar doppler. Int Arch Otorhinolaryngol.

[66] Almotairi FS, Andersson M, Andersson O, Skoglund T, Tisell M (2018). Swallowing Dysfunction in Adult Patients with Chiari I Malformation. J Neurol Surg B Skull Base.

[67] Yu T, Li J, Wang K, Ge Y, Jiang AC, Duan LP (2017). Clinical characteristics of neurogenic dysphagia in adult patients with Chiari malformation type I. Beijing Da Xue Xue Bao Yi Xue Ban.

[68] Lee HH, Seo HG, Kim KD, Lee SH, Lee WH, Oh BM (2018). Characteristics of Early Oropharyngeal Dysphagia in Patients with Multiple System Atrophy. Neurodegener Dis.

[69] Sulena GD, Sharma AK, Singh B (2017). Clinical Profile of Dysphagia in Patients with Parkinson's Disease, Progressive Supranuclear Palsy and Multiple System Atrophy. J Assoc Physicians India..

[70] Rönnefarth M, Hanisch N, Brandt AU, Mähler A, Endres M, Paul F (2020). Dysphagia Affecting Quality of Life in Cerebellar Ataxia-a Large Survey. Cerebellum.

[71] Müller J, Wenning GK, Verny M, McKee A, Chaudhuri KR, Jellinger K (2001). Progression of dysarthria and dysphagia in postmortem-confirmed parkinsonian disorders. Arch Neurol.

[72] Umemoto G, Furuya H, Tsuboi Y, S. F, Arahata H, M. S, (2017). Dysphagia in Multiple System Atrophy of Cerebellar and Parkinsonian Types. Journal of Neurology and Neuroscience.

[73] Poorjavad M, Derakhshandeh F, Etemadifar M, Soleymani B, Minagar A, Maghzi AH (2010). Oropharyngeal dysphagia in multiple sclerosis. Mult Scler.

[74] Thomas FJ, Wiles CM (1999). Dysphagia and nutritional status in multiple sclerosis. J Neurol.

[75] Takizawa C, Gemmell E, Kenworthy J, Speyer R (2016). A Systematic Review of the Prevalence of Oropharyngeal Dysphagia in Stroke, Parkinson's Disease, Alzheimer's Disease, Head Injury, and Pneumonia. Dysphagia.

[76] Humbert IA, McLaren DG, Kosmatka K, Fitzgerald M, Johnson S, Porcaro E (2010). Early deficits in cortical control of swallowing in Alzheimer's disease. J Alzheimers Dis.

[77] Ando S, Kanazawa M, Onodera O (2020). Progressive Supranuclear Palsy with Predominant Cerebellar Ataxia. J Mov Disord.

[78] Clark HM, Stierwalt JAG, Tosakulwong N, Botha H, Ali F, Whitwell JL (2020). Dysphagia in Progressive Supranuclear Palsy. Dysphagia.

[79] Bede P, Elamin M, Byrne S, McLaughlin RL, Kenna K, Vajda A (2015). Patterns of cerebral and cerebellar white matter degeneration in ALS. J Neurol Neurosurg Psychiatry.

[80] Solazzo A, Monaco L, Vecchio LD, Reginelli A, Iacobellis F, Capasso R (2014). Earliest videofluoromanometric pharyngeal signs of dysphagia in ALS patients. Dysphagia.

[81] Teismann IK, Warnecke T, Suntrup S, Steinsträter O, Kronenberg L, Ringelstein EB (2011). Cortical processing of swallowing in ALS patients with progressive dysphagia–a magnetoencephalographic study. PLoS ONE.

[82] Chen Y, Kumfor F, Landin-Romero R, Irish M, Hodges JR, Piguet O (2018). Cerebellar atrophy and its contribution to cognition in frontotemporal dementias. Ann Neurol.

[83] Alagiakrishnan K, Bhanji RA, Kurian M (2013). Evaluation and management of oropharyngeal dysphagia in different types of dementia: a systematic review. Arch Gerontol Geriatr.

[84] Koga S, Josephs KA, Ogaki K, Labbé C, Uitti RJ, Graff-Radford N (2016). Cerebellar ataxia in progressive supranuclear palsy: An autopsy study of PSP-C. Mov Disord.

[85] Flowers HL, Skoretz SA, Streiner DL, Silver FL, Martino R (2011). MRI-based neuroanatomical predictors of dysphagia after acute ischemic stroke: a systematic review and meta-analysis. Cerebrovasc Dis.

[86] Dehaghani SE, Yadegari F, Asgari A, Chitsaz A, Karami M (2016). Brain regions involved in swallowing: Evidence from stroke patients in a cross-sectional study. J Res Med Sci.

[87] Mo SJ, Jeong HJ, Han YH, Hwang K, Choi JK (2018). Association of Brain Lesions and Videofluoroscopic Dysphagia Scale Parameters on Patients With Acute Cerebral Infarctions. Ann Rehabil Med.

[88] Fernández-Pombo A, Seijo-Raposo IM, López-Osorio N, Cantón-Blanco A, González-Rodríguez M, Arias-Rivas S (2019). Lesion location and other predictive factors of dysphagia and its complications in acute stroke. Clin Nutr ESPEN.

[89] Casula EP, Pellicciari MC, Ponzo V, Stampanoni Bassi M, Veniero D, Caltagirone C (2016). Cerebellar theta burst stimulation modulates the neural activity of interconnected parietal and motor areas. Sci Rep.

[90] Nowak DA, Topka H, Timmann D, Boecker H, Hermsdörfer J (2007). The role of the cerebellum for predictive control of grasping. Cerebellum.

